# Cinnamon as a Useful Preventive Substance for the Care of Human and Plant Health

**DOI:** 10.3390/molecules26175299

**Published:** 2021-08-31

**Authors:** Jolanta Kowalska, Józef Tyburski, Kinga Matysiak, Magdalena Jakubowska, Joanna Łukaszyk, Joanna Krzymińska

**Affiliations:** 1 Department of Organic Agriculture and Environmental Protection, Institute of Plant Protection—National Research Institute, Władysława Węgorka 20, 60-318 Poznań, Poland; J.Lukaszyk@iorpib.poznan.pl (J.Ł.); J.Krzyminska@iorpib.poznan.pl (J.K.); 2 Department of Agroecosystems and Horticulture, University of Warmia and Mazury in Olsztyn, Michała Oczapowskiego 2, 10-719 Olsztyn, Poland; jozef.tyburski@uwm.edu.pl; 3 Department of Weed Science and Plant Protection Techniques, Institute of Plant Protection—National Research Institute, Władysława Węgorka 20, 60-318 Poznań, Poland; k.matysiak@iorpib.poznan.pl; 4 Department of Monitoring and Signalling of Agrophages, Institute of Plant Protection—National Research Institute, Władysława Węgorka 20, 60-318 Poznań, Poland; M.Jakubowska@iorpib.poznan.pl

**Keywords:** basic substance, human health, insecticidal activity, microbial activity, plant diseases

## Abstract

Cinnamon is widely used as a food spice, but due to its antibacterial and pharmacological properties, it can also be used in processing, medicine and agriculture. The word “Cinnamon” can refer to the plant, processed material, or an extract. It is sometimes used as a substance, and sometimes used as a mixture or as compounds or a group. This article reviews research into the effectiveness of various forms of cinnamon for the control of plant diseases and pests in crops and during storage of fruit and vegetables. Cinnamon acts on pests mainly as a repellent, although in higher doses it has a biocidal effect and prevents egg-laying. Cinnamon and its compounds effectively hinder bacterial and fungal growth, and the phytotoxic effects of cinnamon make it a possible herbicide. This article presents the wide practical use of cinnamon for various purposes, mainly in agriculture. Cinnamon is a candidate for approval as a basic substance with protective potential. In particular, it can be used in organic farming as a promising alternative to chemical pesticides for use in plant protection, especially in preventive treatments. The use of natural products is in line with the restriction of the use of chemical pesticides and the principles of the EU’s Green Deal.

## 1. Introduction

Cinnamon (*Cinnamomum zeylanicum* L. and *Cinnamon cassia* L.), a species of the *Lauraceae* family, is an evergreen tree of the tropics, which is widely used in medicine, and offers a rich variety of applications worldwide. The word was adopted by English towards the end of 14th century as a loanword from the old French “cinnamone”, which in term came from Latin via the Greek-Phoenician word “kinnamomon” and supposed to be from Semitic cf. Hebrew “qinamon”. The first appearance of the word in print dates to 1430, in John Lydgate Bochas’ “Fall of Princes” [1].

Cinnamon contains manganese, iron, dietary fibre, and calcium. It has derivatives, such as cinnamaldehyde (CNAD), cinnamic acid, cinnamate, and many other ingredients, such as polyphenols and antioxidants, with anti-inflammatory, antidiabetic, antimicrobial, and anticancer properties. Several reports have shown numerous properties of cinnamon in the form of bark and bark powder. Essential oils and phenolic compounds in cinnamon contribute positively to human health. Studies have recently shown the positive influence of cinnamon in the treatment of Alzheimer’s disease, diabetes, arthritis, and arteriosclerosis [2].

Wang et al. [3] reported other major compounds in cinnamon: coumarin, cinnamyl alcohol, cinnamaldehyde, cinnamic acid, eugenol, and cinnamyl acetate [3]. Tung et al. [4] have also reported the presence of a wide range of essential oils in cinnamon, such as *trans*-cinnamaldehyde, cinnamyl acetate, eugenol, l-borneol, caryophyllene oxide, b-caryophyllene, l-bornyl acetate, E-nerolidol, α-cubebene, α-terpineol, terpinolene, and α-thujene. Cinnamon consists of a variety of resinous compounds (Table 1).

According to other sources, ground cinnamon contains carbohydrates, fibre, moisture, protein, fat, and ash. It also contains vitamins A, B, C, E, K, and lipids. The composition is different depending on the geographical origin and the processing methods [6,7].

As a plant, cinnamon contains many substances and substance groups. Among these, there are essential oils, diterpenes, catechins, proanthocyanidins, tanning agents, colouring agents, phenolic carboxylic acids, lignans, and mucins. Cinnamon's essential oils mainly have antifungal and antibacterial properties and, similarly to cinnamon bark extract, are characterized by antioxidant activity [8]. Moreover, essential oils have antiinflammatory, antidiabetic, antitumor, antimutagenic, and memory-enhancing properties. Cinnamaldehyde and eugenol are active components against Gram-positive and Gram-negative bacteria [9].

Sharifi-Rad et al., 2021 [10] showed that the bioactive compounds of *Cinnamomum* species possess antimicrobial, antidiabetic, antioxidant, anti-inflammatory, anticancer, and neuroprotective effects.

Incomplete knowledge about the safe consumption of higher doses of cinnamon on a daily basis makes it necessary to assess the occurrence of this risk, and therefore, the long-term use of a high amount of cinnamon should be monitored. The tolerable daily intake for coumarin (0.1 mg/1 kg body weight) can be regarded as safe in terms of daily cinnamon intake without the risk of adverse effects [11]. According to the scientific data currently available, a risk assessment must be focused on the problematic ingredients of cinnamon extract, especially on coumarin, *trans*-cinnamaldehyde, safrol, and styrene, which are toxic.

Cinnamon bark is obtained twice a year, closely following each of the rainy seasons, when the air humidity facilitates bark harvesting. The first harvest is done when the trees are three years old, a year after pruning. The side stems that are about three-years-old are cut off, and the bark is pulled off. Cinnamon bark is gained only from stems that are between 1.2 and 5 cm in diameter.

Cinnamon is often ground to a powder before sale. The powder should be packed in moisture-proof wrapping (polypropylene bags) to keep the flavour. Polyethylene packaging is not advisable, as the flavour components diffuse through it [12].

## 2. Culinary and Medicinal Use

Cinnamon bark is commonly used as a spice. It is principally used in cooking as a condiment and flavouring agent. It is used in the production of chocolate, especially in Mexico, which is the biggest importer of true cinnamon (*C. zeylanicum* L.). It is also added to desserts, such as apple pie, donuts, and cinnamon buns, as well as spicy candies, tea, hot cocoa, and liqueurs. True cinnamon and not cassia (*C. cassia* L.) is better for use in sweet dishes. In the Middle East, it is often used in savoury dishes of chicken and lamb. In the USA, cinnamon is often used as an additive to flavour cereals, bread-based dishes, and fruit, especially apples; and a cinnamon–sugar mixture is on sale in grocery stores. Another use of cinnamon is in pickling.

Cinnamon bark is one of the rare spices that can be consumed directly; cinnamon powder has long been an important spice in Persian cuisine, added to various thick soups, drinks, and sweets. It is often used as a mixture with rosewater or other spices to make a cinnamon-based curry for stews or just sprinkled on sweet desserts [13].

### 2.1. Effects in Humans

Cinnamaldehyde (CNAD) lowers inflammatory reactions, oxidative stress, and apoptosis of the liver in *Salmonella typhimurium*-infected mice. Supplementation of CNAD might be a good preventive method to alleviate the liver damage caused by *Salmonella typhimurium* infection in humans and animals [14]. Moreover, cinnamon bark essential oils (EOs) have been shown to cause oxidative stress to *Klebsiella pneumoniae*, resulting in the loss of cell viability [15]. Both oregano and cinnamon bark EOs have strong antibacterial properties. Aljaafari et al. [16] have shown that the antimicrobial properties of essential oils (EOs) are based on the mode of action in relation to membrane disruption, efflux inhibition, the increase in membrane permeability, and the decrease in intracellular ATP. These essential oil compounds can be used as potential agents against bacteria, fungi, and viruses. In the future, the integration of EOs uses can lead to many clinical applications.

In medicine, the essential oils in cinnamon behave like other volatile oils. It has also been used in the treatment of digestive system problems and colds. The essential oils in cinnamon also have antimicrobial properties and are used as a preservative in some foods. Cinnamon has been reported to have remarkable pharmacological effects in the treatment of diabetes type 2 resistant to both mellitus and insulin; however, the plant material used in the study was mainly from cassia and only some of the plant material was from *C. zeylanicum*. Cinnamon has traditionally been used for toothache and to fight bad breath, and its regular use is thought to cure the common cold and support digestion [17]. It is noted that regular drinking of *C. zeylanicum* tea made from the bark could be helpful in oxidative-stress-related illness in humans, since it has considerable antioxidant potential. Cinnamon may also act as an aphrodisiac. One teaspoon of cinnamon has as many antioxidants as a cup of pomegranate juice and half a cup of blueberries [17].

Nanocapsules with cinnamon-thyme-ginger composite essential oils prepared with chitosan as the wall via ionic gellification reaction were tested in medicine and revealed durable antibacterial activity against *Escherichia coli*, *Bacillus subtilis*, and *Staphylococcus aureus*. Composite essential oil nanocapsules CEO-NPs can be applied as a strong long-lasting natural preservative [18].

### 2.2. Adverse Effects Reported in Humans 

Scientific research has confirmed the effectiveness of cinnamon in fighting microbes, viruses, fungi, oxidants, tumours, and hypertension. It also has antidiabetic, gastro-protective, and immune modulatory potential [17]. However, the popular use of cinnamon has also resulted in several reports of side effects from its short- and long-term use. The most common negative effects were disorders of the stomach and bowels, as well as allergic reactions, which were self-controlling in most cases. Although cinnamon is safe as a spice and/or flavour, prolonged and enlarged use may be a health risk, and hence, in medicinal uses, it should be clinically monitored [19].

Cinnamon coats and dries the mouth and throat, leading to coughing, gagging, vomiting, and the inhalation of cinnamon, causing throat irritation, breathing difficulties, and a risk of pneumonia or lung collapse [20,21]. Cinnamon contact stomatitis (CCS) is also a sporadic reaction to the consumption of foods containing artificial cinnamon flavour. Physicians who treat patients with oral conditions ought to be aware of CCS to correctly diagnose and manage this condition [22]. Contact stomatitis, which is related to the use of cinnamon flavourings, is rather rare. The symptoms, as well as the histopathologic features of this disease are non-specific. They may be similar to other inflammatory illnesses of the oral mucosa, which causes problems in diagnosis. Patients develop white and erythematous spots of rapid occurrence, with an associated sensation of burning. High levels of coumarin and cinnamaldehyde might be associated with mouth sores [23]. High levels of coumarin and cinnamaldehyde might be also correlated to liver damage and low blood sugar [24]; such high levels may increase the risk of cancer, breathing problems, and interaction with certain medications [11].

Oral lesions caused by a reaction to cinnamon flavouring agents are rather uncommon and are probably unrecognized by many physicians. Most patients feel a “burning sensation”, which is the primary symptom. Clinically, lesions present as erythematous patches with different degrees of superimposed keratosis or ulceration. The lesions are usually limited to the buccal mucosa and lateral border of the tongue. The responsible agent was most frequently cinnamon-flavouring chewing gum, and symptoms usually eased within 1 to 2 days after the last use of the product containing cinnamon [25].

## 3. Agricultural Purpose

### 3.1. Fungicidal Activity

Cinnamon oils and extracts possess antifungal properties against serious plant diseases (Table 2). Wilson et al. [26] indicated that, out of 49 essential oils tested, cinnamon leaf *C. zeylanicum* showed the strongest antifungal activity against *Botrytis cinerea*. Montes-Belmont and Carvajal [27] found that *Aspergillus flavus* was fully inhibited by *C. zeylanicum*. In other studies, *C. zeylanicum* proved to be fungicidal towards pathogens (*Colletotrichum musae*, *Lasiodiplodia thebromae*, and *Fusarium proliferatum*) isolated from bananas [28]. Cinnamon also had an antifungal effect against *Oidium murrayae* [29] and harnessed conidial germination of *Colletotrichum gloesporioides* [30]. In in vitro tests, it proved to have a significant mycelial inhibition in corn rot *Fusarium oxysporum* f. sp. *gladioli* [31] and to be very effective against the growth of *Rhizoctonia solani* [32]. Moreover, cinnamon has powerful antifungal activity towards early tomato blight (*Alternaria solani*) [33]. *Botrytis cinerea* is a serious problem, especially in horticultural crops. The investigations of Wang et al. [34] demonstrated that cinnamon microemulsions possessed high in vivo control properties against gray mould of pears, *B. cinerea.* The influence of *C. zeylanicum* organic powder on the growth of *B. cinerea* and its effect on tomato plants have also been assessed. Cinnamon bark powder and also its water suspensions and filtrates controlled *B. cinerea*; moreover, tomato plants sprayed with cinnamon developed better than the control plants [35].

An in vitro study on extracts from the cinnamon bark of *C. cassia* and clove (*Syzygium aromaticum* L.) on the growth of *B. cinerea* colonies showed their antifungal activity, causing a slow growth of this pathogen and can be applied to control strawberry gray mould. The clove extract consisted of 52.88% eugenol, and the cinnamon extract consisted of 74.67% cinnamaldehyde. The efficacy of the extracts on detached strawberry leaves showed that a 12 mL/L concentration of clove extract was effective in suppressing grey mould infection. It is worth noting that the results indicate that the antifungal properties of the clove extract were more effective (even applied in a lower concentration) than that of the cinnamon extract. Grey mould infection on detached strawberry leaves was inhibited by the use of clove oil at the higher investigated concentration (12 mL/L) [36]. The cinnamon extract applied at a rate of 12 mL/L proved to be less effective at inhibiting the spread of grey mould on strawberry leaves [37], and Allam et al. [38] reported that a higher concentration of 20 mL/L of cinnamon entirely inhibited the mycelial growth of *B. cinerea* in vitro.

Cinnamaldehyde has been used effectively as a bio-fungicide for controlling other plant fungal diseases. For example, it showed good inhibitory activity against *Colletotrichum lagenarium*, a significant plant-pathogenic fungus leading to anthracnose of cucumber. Other studies indicate that it markedly inhibited zoospore germination and the rapid mycelial development of *Phytophthora capsici*, a pathogenic fungus causing phytophthora blight of pepper [39]. Essential oil extracted from *C. zeylanicum* (CEO) leaves was identified as having the active constituents eugenol and *trans*-cinnamaldehyde, which had, respectively, minimal inhibitory concentrations (MICs) of 250 and 62.5 μg/mL against *Alternaria alternata*, while, under the same conditions, the MICs for a commercial fungicide and CEO were 1250 and 500 μg/mL, respectively [40].

Cinnamon essential oil proved to be more capable of limiting the incidence and progress of fungal disease in commercial tangerine orchards than copper fungicide and was effective at a similar level as a commercial plant activator. Both essential oil and cinnamaldehyde (additional to the direct way of action, inhibiting fungal growth), positively influence the plant defense system, causing a significant rise in enzyme levels [40]. It was conferred that cinnamon oil has powerful antifungal activity against these four species of fungi: *Aspergillus niger*, *Penicillium notebookum*, *Mucora heimalis,* and *Fusarium oxysporum*. Cinnamon oils and cinnamon extracts have demonstrated good antifungal properties against economically important plant diseases [41].

The effectiveness of essential oil from clove and cinnamon against fungi resulting in the postharvest decay of grapes: *A. niger*, *A. alternata*, *Colletotrichum gloeosporioides*, *L. theobromae*, *Phomopsis viticola*, and *Rhizopus stolonifer* has also been investigated. In the study of Udomlak Sukatta [42] the antifungal activity of clove oil against all the above-mentioned fungi showed minimal inhibitory concentrations (MICs) of: 200, 200, 400, 800, 200, and 200 mg/mL, respectively, whereas the MICs obtained from cinnamon oil were 50, 100, 200, 200, 100, and 800 mg/mL, respectively. Investigation of the synergistic effect of clove and cinnamon oil showed three optimum ratios: 3:7, 2:8, and 1:9 and MICs for all fungi obtained from these ratios for the inhibition of the growth of six fungi was 400 mg/mL. A study of the synergistic effect of clove and cinnamon oil indicated three optimum proportions: 3:7, 2:8, and 1:9, and the MICs for all fungi obtained from these ratios to inhibit the development of six fungi was 400 mg/ mL [42].

A further study determined if essential oils can be applied as a contact fungicide seed treatment for organic maize. The sowing date for organic maize (*Zea mays* L.) is delayed to avoid the coldness and wetness of spring soils. Conventional farmers can apply chemical fungicide seed treatments to protect the emerging seedling but almost no organic fungicides are on the market. Eighteen plant essential oils were studied for their fungicidal activities against three common maize pathogens: *Penicillium* spp., *Fusarium* spp., and *Pythium* spp. Five oils fully controlled all three pathogens in vitro. These oils were cinnamon, clove, oregano, savory, and thyme. The MIC for all pathogens was 800 µL/L, and no phytotoxicity was detected in the germination test at doses up to 16,000 µL/L (MIC × 20). The field emergence of inbred and hybrid seeds treated with the essential oil were considerably decreased compared to seed treated with the commercial, conventional fungicides and one organic fungicide [43].

Fusarium wilt caused by *F. oxysporum* f. sp. *lycopersici* is the most serious disease of the tomato. In the study of Prashant et al. [44], seven plant extracts were screened against *F. oxysporum.* Among these, the antifungal properties of *S. aromaticum* and *C. zeylanicum* extracts were comparable to the efficiency of chemical fungicides. Methanol (MeOH) extract of *S. aromaticum* demonstrated the widest range of inhibition as compared to other extracts assessed, including those from *C. zeylanicum*. Solvent extracts of cinnamon and clove proved to be 100% inhibitory against *F. oxysporum* spores at 5 and 10 mL/L rates [44].

Another pathogen, *Fusarium verticillioides,* is a filamentous fungus and a commonly occurring pathogen with the ability to infect and destruct economically important crops by producing fumonisin mycotoxins. Xing et al. [45] carried out a study to assess the inhibitory properties of cinnamon and clove, eucalyptus, anise, spearmint, and camphor oils on *F. verticillioides*. Cinnamon oil proved to have the strongest inhibition properties. The antifungal potential of cinnamon oil was investigated with a special focus on its mechanism of inhibition of *F. verticillioides* development at the morphological and ultra-structural planes. For *F. verticiliides*, the minimal inhibitory concentrations (MICs) of cinnamon oil (85% cinnamaldehyde), natural cinnamaldehyde (95%), and synthetic cinnamaldehyde (99%) were 60, 50, and 45 mL/L, respectively. The same authors [45] used scanning electron microscopy and its transmission version of *F. verticillioides* in the presence of MIC of cinnamaldehyde and showed irreversible deleterious morphological and ultra-structural changes, such as a lack of cytoplasmic content, depravation of integrity and rigidity of the cell wall, plasma membrane disintegration, mitochondrial destruction, and the folding of the cell. These alterations caused by cinnamaldehyde may result from its interference with the enzymatic reactions of cell wall synthesis, thus negatively influencing the morphogenesis and development of the fungus. These outcomes further emphasized the toxicity of cinnamon oil towards *F. verticillioides* attacking grains. It shows that cinnamon oil can be safely applied as an alternative to chemical fungicides during grain storage. The cinnamon oil concentration proved to be effective on the development of *F. verticillioides* with fumigation. The inhibitory effect of cinnamon oil rose proportionally to its concentration and proportionally to treatment duration. An increase in cinnamon oil concentration resulted in a delay in conidia germination and showed different inhibitory reactions. At a rate of 40 mL per Petri dish after 6 days of incubation, the cinnamon oil fully inhibited *F. verticillioides* mycelial growth. After 20 days of incubation, visible development of *F. verticillioides* did not take place. These results indicate the fungistatic properties of cinnamon oil at lower ratios and fungicidal properties at higher ratios [45]. The ability of cinnamon, clove, lemongrass, oregano, and palmarosa essential oils to prevent the growth of fumonisin B1 (FB1) production by *F. verticillioides* at different water activities (0.95 and 0.995 aw) and temperatures (20 and 30 °C) in irradiated maize grain was also evaluated by Velluti et al. [46]. All of the essential oils inhibited growth of *F. verticillioides* isolates under all conditions tested, but FB1 production was only inhibited at 30 °C and 0.995 aw.

The antifungal properties of cinnamon against other pathogens have been shown by other researchers [47]. The in vitro efficacy of cinnamon essential oil was tested at application rates of 100, 250, 500, 1000, and 2000 µL/L for controlling fruit rot. The mycelium growth of *Colletotrichum gloeosporioides* sp., *Fusarium solani*, and *Phytophthora palmivora* was inhibited at application rates of 1000 µL/L [47].

The antifungal effect of cinnamon oil has been studied with special reference to its mechanism of inhibition on *F. verticillioides* growth at the morphological and ultra-structural levels. The MICs of cinnamon oil (85% cinnamaldehyde), natural cinnamaldehyde (95%), and synthetic cinnamaldehyde (99%) were 60, 50, and 45 μL/L, respectively. The antifungal activity of cinnamon oil was proportional to its cinnamaldehyde concentration [45].

A significant antifungal effect was observed with the essential oil of C. *zeylanicum* on mycelial growth using bioassays of *Fusarium oxysporum* f. sp. *gladioli* at 100, 150, 200, 250, and 300 ppm [31]. The important antifungal potential of cinnamon oil (both in vitro and in vivo) in proportion to its concentration towards various *Fusarium* species was confirmed. In in vitro studies by Horváth et al. [48], the cinnamon oil effectively controlled mycelial development of Fusarium head blight of winter wheat. Jiang et al. [49] demonstrated that *C. cassia* oil has a significant antifungal effect against *S. sclerotiorum* with a minimum inhibitory concentration (MIC) of 256 μg/mL in agar and 64 μg/mL in air. In a further study, *trans*-cinnamaldehyde exhibited the highest antifungal activity among the three cinnamaldehydes tested. Al-Taisan et al. [50] noted that cinnamon, clove, and mint oils completely inhibited in vivo mycelial growth of *S. sclerotiorum* at 10–500 ppm concentrations. A soil application containing cinnamon oil significantly reduced the incidence of disease caused by *S. sclerotiorum*, producing 75% plant survival compared to the control [43]. Moraes et al. [51] investigated the inhibitory properties of cinnamon (*C. cassia*) and citronella (*Cymbopogon winterianus*) essential oils in the in vitro control of *Aspergilus* sp. and *S. sclerotiorum* fungi. The effectiveness of cinnamon and other essential oils and microelements against *Sclerotinia sclerotiorum* was shown in in vitro tests. Cinnamon and citronella essential oils were used in doses of 0.2, 0.4, 0.8, and 1.6 mL/L. The dose of 1.6 mL/L of both oils fully inhibited the mycelial development of *Aspergillus* sp. and *S. sclerotiorum* fungi.

The continuous spread and evolution in the development of natural plant protective means as alternatives to synthetic fungicides attracts attention today. Combrinck et al. [52] evaluated the antifungal properties of eighteen essential oils and their impact on the growth of five pathogens in vitro (*Lasiodiplodia* *theobromae*, *Colletotrichum gloeosporioides*, *Alternaria citrii*, *B. cinerea*, and *Penicillium digitatum*) isolated from mango, avocado, citrus, grapes, and cactus pear. Most of the oils were chosen because of their commercial availability and the content of a predominant compound. A visual inspection of the fungal growth was conducted, and the lowest ratio where the fungal growth was fully stopped was noted. Rich in eugenol (81.2%), cinnamon oil showed a strong fungicidal effect [45]. In other research, the antifungal properties of cinnamon extract (CE) were assessed on banana crown rot fungi. The antifungal activities of cinnamon extract, pepper extract (PE), and garlic extract (GE) were evaluated on banana crown rot fungi (*Colletotrichum musae*, *Fusarium* spp., and *L*. *theobromae*) in vitro. The assay was conducted with extracts of CE, PE, and GE with concentrations of 0, 0.1, 0.5, 1.0, 5.0, 10.0, and 0.75 g/L of carbendazim (CBZ) on potato dextrose agar at room temperature. CE completely inhibited the conidial germination and mycelial growth of all three fungi at 5.0 g/L. PE totally suppressed mycelial growth of all fungi at 5.0 g/L and conidial germination at 10.0 g/L except for Fusarium spp. GE had no significant effects, but low concentrations (0.1 and 0.5 g/L) enhanced germ tube elongation of the three fungi. Crown rot growth was also evaluated during banana storage at 13 °C for 7 weeks. The disease development was weakest (25%) on CE-treated fruit after inoculation and stronger when CE was applied prior inoculation. CE had no negative effects on the quality of the fruit, but CE used together with hot water treatment led to unacceptable skin browning [53]. The aim of another study was to assess the antifungal potential of crude extracts of cinnamon and rosemary against three isolates of sclerotinia carrot rot both under in vitro and in vivo conditions. The extracts were obtained by applying two different solvents with ethyl acetate (EA) and ethanol. The results indicated that crude extracts of cinnamon can reduce the mycelial development of one isolate at the volatile and contact phase by 35.4% and 78.2%, respectively. Although crude extracts of cinnamon and rosemary could lower the severity of carrot rot during carrot storage in contrast to EA, an extract of cinnamon (2 g/L) was confirmed to have a significant effect against this disease [54].

**Table 2:** Selected examples of fungicidal activity of cinnamon components for agricultural purposes.

Pathogen	Form of Cinnamon	Effective Dose	Reference No.
*Fusarium oxysporium*	Essential oil	100–300 ppm	[31]
*Botrytis cinerea*	Extract *C. cassia*	20 mL/L	[38]
*Fusarium verticillioides*	Essential oil Cinnamon oil with cinnamaldehyde	60 µL/L 45–60 µL/L	[45]
*Colletotrichum gloerpoides* *Phytophthora palmivora* *Fusarium solani*	Essential oil	1000 µL/L	[47]
*Sclerotinia sclerotiorum*	Essential oil	10–500 ppm	[50]
*Sclerotinia sclerotiorum*	*C. cassia* oil	256 µg/mL of agar	[51]
*Sclerotinia scleriotiorum* *Aspergillus* sp.	*C. cassia* oil	1.5 mL/L	[51]
*Lasidiploidia theobromae* *Colletotrichumgloerpoides* *Alternaria citrii*	Essential oil	1000 µL/L	[54]
*Colletotrichum musae* *Lasidiploidia theobromae*	Cinnamon extract	5 g/L	[55]
*Sclerotinia sclerotiorum*	ethyl acetate cinnamon extract	2 g/L	[56]

Mohammed et al. [55] studied the potential of *E*-cinnamaldehyde (EC) against *S. sclerotiorum* on potatoes and also the induction of glutathione *S*-transferase genes. The findings indicated that EC could inhibit the mycelial growth of *S. sclerotiorum. E*-cinnamaldehyde decreased white mould on potatoes in greenhouse trials; additionally, *E*-cinnamaldehyde considerably strengthened the activity of glutathione *S*-transferase (GST)-like genes identified from the pathogen genome [55].

Another in vitro study indicated the antifungal activity of five plant extracts, including *C. zeylanicum*, conducted with either cold distilled water (CDW) or boiling water (BDW) on two pathogenic fungi, *A. alternata* and *F. oxysporum.* The results indicate that plant extracts, especially those treated with CDW, revealed a powerful antifungal activity with significant inhibition of the development of the two tested fungi [56].

### 3.2. Bactericidal Activity

Imad et al., 2016 [57] established that *C. zeylanicum* possesses remarkable antimicrobial activity, which is predominantly due to *E*-cinnamaldehyde. These findings indicate that the methanolic extract of *C.* *zeylanicum* is antifungal and antibacterial. Natural biocides with *C. zeylanicum* bark essential oil have major potential as antimicrobials; this potential is reduced due to volatility and the fast decomposition of the essential oil. To prevent this and to lengthen the efficacy of this biocide, cinnamaldehyde (CNAD) was encapsulated into mesoporous silica nanoparticles (MSNPs) in order to handle the problem. To eliminate seedborne diseases, CNAD-MSNPs were dressed in a sodium alginate seed coating. This system was tried against *Pseudomonas syringae* pv. *pisi*, responsible for pea bacterial blight. However, the concentration of CNAD in the alginate coating was <0.0000034% (*v/v*); this was up to 90,000-fold lower than the concentrations of free cinnamon oil reported earlier to control some bacterial diseases [58].

### 3.3. Insecticidal Activity

Cinnamon has been suggested for use as a repellent against insects. Cinnamon oils and their components, such as cinnamaldehyde, are insecticidal compounds that have been used against a variety of insects [41] (Table 3).

Cinnamon leaf oil proved to be very effective as a killing agent for mosquito larvae. The compounds cinnamaldehyde, cinnamyl acetate, eugenol, and anethole, which are ingredients of cinnamon leaf oil, were found to be the most effective against mosquito larvae. The insecticidal and fumigant properties of *C. cassia* bark-derived materials against the oak nut weevil (*Mechoris ursulus*) were examined using filter paper diffusion and fumigation methods. In a test with the filter paper diffusion method, *trans*-cinnamaldehyde showed 100 and 83.3% mortality at rates of 2.5 and 1.0 mg/filter paper, respectively. At 2.5 mg/paper, strong insecticidal activity was produced from eugenol (90.0% mortality) and salicylaldehyde (88.9%), whereas *trans*-cinnamic acid had a moderate activity (73.3%). At 5 mg/paper, weak insecticidal activity (50.0%) was produced from cinnamyl alcohol. In a fumigation study, the cinnamon bark-derived components were considerably more effective in closed cups than in open ones. The results indicated that the insecticidal activity of the tested compounds was attributable to fumigant action, but significant contact toxicity also occurred [59].

Cinnamon oil has been applied to control thrips on onions. The effects of orange and cinnamon oil (from *C. zeylanicum*) on the presence and damage of *Thrips tabaci* on onions was studied. The results (expressed as the number of thrips caught with sweeping nests) confirm that both orange and cinnamon oil considerably reduced the number of adults on onion plants [60]. To test the controlling potential against agricultural pests, such as the diamondback moth (*Plutella xylostella*), green peach aphid (*Myzus persicae*), and two spotted spider mite (*Tetranychus urticae*), the essential oils of *Coriandrum sativum* and *C. cassia* obtained from steam distillation, hexane extraction, and supercritical extraction were assessed. Using the contact bioassay, the LD_50_ values of *C. cassia* oils prepared by steam distillation and hexane extraction methods were 5.96 and 4.64 µg/cm^2^, respectively, against *T. urticae* adults, and the LD_50_ value of the essential oil by the supercritical extraction method was 6.50 µg/cm^2^ against *M. persicae* adults. Finally, the research indicated that *C. cassia* oils are a promising natural acaricide and insecticide against pests [61].

In other studies the toxicity and deterrence of the terpenoid constituents of cinnamon oil have been evaluated. The toxic effects, repellent properties, and respiration rate influenced by terpenoid components of cinnamon essential oil were assessed, as well their influence on *Sitophilus granarius* L. The lethal concentrations (LC_50_ and LC_90_), repellence in general, and behaviour repellence reaction of adult *S. granarius* in the presence of six concentrations of respective essential oils, as well as terpenoids, were assessed. The chemical constituents of the cinnamon oil were established, and its primary compounds were eugenol (10.5%), *trans*-3-caren-2-ol (10.2%), benzyl benzoate (9.99%), caryophyllene (9.34%), eugenyl acetate (7.71%), α-phellandrene (7.41%), and α-pinene (7.14%). The toxicity of cinnamon essential oil against *S. granaries* was showed. Among the toxic terpenoid components, eugenol has the most powerful contact toxicity against *S. granarius* in comparison to, in decreasing order, caryophyllene oxide, α-pinene, α-humulene, and α-phellandrene. Cinnamon essential oils, as well as their terpenoid compounds, proved toxic and had repellent properties against adult *S. granarius* and thus have preventive and retarding properties for the development of resistance to insecticide [62].

In another study with storage pests, the bean weevil *Acanthoscelides obtectus*, which is the cause of heavy post-harvest losses in the common bean, *Phaseolus vulgaris*, the essential oil of *C. zeylanicum* was tested for insecticidal (lethal toxicity, disturbances to reproduction, and persistence of action) and repellent activities. The study revealed toxicity at LD_50_ 46.8 μL/kg beans, steadily reduced the growth rate of *A. obtectus* in a dose-related manner, and showed a similar loss of its insecticidal potential with time. Cinnamon oil also repelled the bean weevil. The results showed that cinnamon essential oil is an effective tool for protecting stored beans against *A. obtectus* in small storage facilities. Cinnamon oil inhibited the reproduction ability of the flour beetle (*Tribolium castaneum*), the maize weevil (*Sitophilus zeamais*), and the lesser grain borer (*Rhyzopertha dominica*) at a rate of 0.1–0.2%, mixed with wheat or wheat flour [63].

The insecticidal properties towards adults of *S. oryzae* L. and *Callosobruchus chinensis* were tested by Soon-Il Kim et al. [64]. In a test with a filter paper diffusion method at 3.5 mg/cm^2^, an extract from *C. cassia* bark and oil was used. An extract from *C. sieboldii* root bark gave 100% mortality at two days after treatment. At 0.7 mg/cm^2^, extracts from *C. cassia, C. sieboldii,* and cinnamon oil were highly effective against both species. In a fumigation test with *S. oryzae* adults, the oils were much more effective in closed containers than in open ones, indicating that the insecticidal activity of the oils was attributable to fumigant action. The authors concluded that these plant extracts and essential oils could be helpful in controlling field populations of *S. oryzae* and *C. chinensis*. In another paper on *Cydalima perspectalis,* the results point to cinnamon oil as a good deterrent with the strongest oviposition-deterring effect [65].

### 3.4. Nematicidal Activity

The nematicidal activity of *C. cassia* and *C. zeylanicum* oils (bark and green leaf) and their chemical compounds against adult *Bursaphelenchus xylophilus* was tested by a direct contact bioassay. The LC_50_ values for cassia oils (0.084–0.085 mg/mL) and for cinnamon oils (0.064–0.113 mg/mL) were toxic towards adult *B. xylophilus*. Of 45 tested compounds, *trans*-cinnamaldehyde (0.061 mg/mL) was the most active nematicide, followed by ethyl cinnamate, α-methyl-*trans*-cinnamaldehyde, methyl cinnamate, and allyl cinnamate (0.114–0.195 mg/mL). Potent nematicidal activity was also observed with 4-methoxycinnamonitrile, *trans*-4-methoxycinnamaldehyde, *trans*-2-methoxy-cinnamaldehyde, ethyl α-cyanocinnamate, cinnamonitrile, and cinnamyl bromide (0.224–0.502 mg/mL). The tested compounds have been described as potential nematicides to combat *B. xylophilus*, which causes pine wilt disease [66].

## 4. Effect of Cinnamon Oil on Plant Growth

The impact of seed dressing with various concentrations of cinnamon oil and tea tree oil on the field emergence and yield of parsley var. Berlinska and lettuce var. Ewelina is presented in the literature [67]. Cinnamon oil lowered the lettuce and parsley field emergence. The toxicity was the greatest at 15% concentration. Tee tree oil showed no toxicity in 55 and 70% concentrations and increased the field emergence ability, particularly in lettuce. A similar relation was established for the speed and spread of emergence. A 15% concentration of cinnamon oil caused a decrease in the number of plants for both species [67].

In laboratory and greenhouse investigations, the allelopathic effect of essential oils extracted from aromatic plants (cinnamon, lavender, and peppermint) on the seed germination of mediterranean weed species (*Amaranthus retroflexus* L., *Solanum nigrum* L., *Portulaca oleracea* L., *Chenopodium album* L., *Sinapis arvensis* L., *Lolium* spp., and *Vicia sativa* L.) was examined. Each essential oil was examined at four concentrations in controlled conditions (germination chamber: 0.2, 0.6, 1.8, and 5.4 mg/L) and in a semi-controlled condition (green house: 5.4, 21.6, 86.4, and 345.6 mg/L). In the controlled conditions, the 1.8 and 5.4 mg/L concentrations stopped seed germination. In the greenhouse (semi-controlled conditions), the 345.6 mg/L concentration of cinnamon essential oil stopped the seed germination of *Amaranthus retroflexus* L. The concentration of essentials oils had a greater effect on weed susceptibility than the type of oil used. However, cinnamon oil had drastic inhibitory effects on germination.

The possible use of essential oils as natural herbicides to control different weeds for the sustainable cropping system has been discussed in relation to their low persistence (due to biodegradability and easy catabolism in the environment); they have no toxicity towards vertebrates, fishes, birds, and mammals and are of importance in plant protection [68].

In conclusion, cinnamon is a suitable candidate for approval as a basic substance with protective potential. In particular, it can be used in organic farming as a promising alternative to chemical pesticides for use in plant protection, especially in preventive treatments. The use of natural products is in line with the restriction on the use of chemical pesticides and the principles of the EU’s Green Deal [69].

## Figures and Tables

**Table 1:** Various chemical constituents of the cinnamon plant [5].

Part of the Plant	Compound
Leaves	Cinnamaldehyde: 1.00 to 5.00%
	Eugenol: 70.00 to 95.00%
Bark	Cinnamaldehyde: 65.00 to 80.00%
	Eugenol: 5.00 to 10.00%
Root bark	Camphor: 60.00%
Fruit	*trans*-Cinnamyl acetate (42.00 to 54.00%) and caryophyllene (9.00 to 14.00%)
Buds (*C. zeylanicum*)	Terpene hydrocarbons: 78.00%
	α-Bergamotene: 27.38%
	α-Copaene: 23.05%
	Oxygenated terpenoids: 9.00%
Flowers (*C. zeylanicum*)	(*E*)-Cinnamyl acetate: 41.98%
	*trans-*α-Bergamotene: 7.97%
	Caryophyllene oxide: 7.20%

**Table 3:** Insecticidal activity of cinnamon.

Insect Pest	Cinnamon Substance/Form	Effective Dose for LC_50_	Reference No.
Mosquito larvae *Aedes aegyptii*	Cinnamon oil *C. cassia*	58.41 mg/L	[59]
*Thrips tabaci*	Essential oil *C. zeylanicum*	Trade product with 70% oil	[60]
*Mechoris ursulus*	*C. cassia* bark extract	2.5–5 mg/paper filter	[59]
*Plutella xylostella* *Tetranychus urticae*	Essential oil *C. cassia*	5.96 µg/cm^2^	[61]
*Myzus persicae*	Essential oil *C. cassia*	6.50 µg/cm^2^	[61]
*Sitophilus granarius*	Essential oil	1000 µL/L	[62]
*Acanthoscelides obtectus*	Essential oil *C. zeylanicum*	46.8 µL/kg beans	[63]
*Tribolium castaneum* *Sitophilus zeamais* *Rhyzopertha dominica*	Essential oil	0.1–0.2% mixed with flour	[63]
*Sitophilus oryzae* *Callosobruchus chinensis*	Extract of *C. sieboldii* root bark, *C. cassia* extract and oil	0.7 mg/cm^2^	[64]
*Cydalima perspectalis*	Cinnamon oil as deterrent	1.5 mL in dispenser	[65]

Source: own study.

## Data Availability

Not applicable.
